# In-stent restenosis is associated with proliferative skin healing and specific immune and endothelial cell profiles: results from the RACHEL trial

**DOI:** 10.3389/fimmu.2023.1138247

**Published:** 2023-05-31

**Authors:** Íñigo Lozano, Roi Bangueses, Isabel Rodríguez, Marta Pevida, Raúl Rodríguez-Aguilar, Diana Rodríguez, Martina Espasandín-Arias, Sara Llames, Álvaro Meana, Ana Suárez, Javier Rodríguez-Carrio

**Affiliations:** ^1^ Department of Cardiology, Hospital Universitario Cabueñes, Gijón, Asturias, Spain; ^2^ Cardiac Pathology Research Group, Instituto de Investigación Sanitaria del Principado de Asturias (ISPA), Oviedo, Asturias, Spain; ^3^ Blood Tansfusion Center and Tissue Bank of Asturias, Centro de Investigación Biomédica en Red de Enfermedades Raras (CIBERER), Oviedo, Asturias, Spain; ^4^ Grupo de Investigación en Oftalmología, Ciencias de la Visión y Terapias Avanzadas (GOVITA), Instituto de Salud del Principado de Asturias (ISPA), Oviedo, Asturias, Spain; ^5^ Instituto Universitario Fernández-Vega, Fundación de Investigación Oftalmológica, Oviedo, Asturias, Spain; ^6^ Department of Pathology Anatomy, Hospital Universitario Cabueñes, Gijón, Asturias, Spain; ^7^ Department of Dermatology, Hospital Universitario Cabueñes, Gijón, Asturias, Spain; ^8^ Centro de Investigación Biomédica en Red en Enfermedades Raras (CIBERER), Instituto de Salud Carlos III, Madrid, Spain; ^9^ Fundación Jiménez Díaz, Madrid, Spain; ^10^ Area of Immunology, Department of Functional Biology, University of Oviedo, Oviedo, Asturias, Spain; ^11^ Grupo de Investigación Básica y Traslacional en Enfermedades Inflamatorias, Instituto de Investigación Sanitaria del Principado de Asturias, Oviedo, Asturias, Spain

**Keywords:** neoatherosclerosis, vascular biology, inflammation, skin healing, restenosis, T cells

## Abstract

**Introduction:**

In-stent restenosis (ISR) is a major challenge in interventional cardiology. Both ISR and excessive skin healing are aberrant hyperplasic responses, which may be functionally related. However, the cellular component underlying ISR remains unclear, especially regarding vascular homeostasis. Recent evidence suggest that novel immune cell populations may be involved in vascular repair and damage, but their role in ISR has not been explored. The aims of this study is to analyze (i) the association between ISR and skin healing outcomes, and (ii) the alterations in vascular homeostasis mediators in ISR in univariate and integrative analyses.

**Methods:**

30 patients with ≥1 previous stent implantation with restenosis and 30 patients with ≥1 stent without restenosis both confirmed in a second angiogram were recruited. Cellular mediators were quantified in peripheral blood by flow cytometry. Skin healing outcomes were analyzed after two consecutive biopsies.

**Results:**

Hypertrophic skin healing was more frequent in ISR patients (36.7%) compared to those ISR-free (16.7%). Patients with ISR were more likely to develop hypertrophic skin healing patterns (OR 4.334 [95% CI 1.044–18.073], p=0.033), even after correcting for confounders. ISR was associated with decreased circulating angiogenic T-cells (p=0.005) and endothelial progenitor cells (p<0.001), whereas CD4^+^CD28^null^ and detached endothelial cells counts were higher (p<0.0001 and p=0.006, respectively) compared to their ISR-free counterparts. No differences in the frequency of monocyte subsets were found, although Angiotensin-Converting Enzyme expression was increased (non-classical: p<0.001; and intermediate: p<0.0001) in ISR. Despite no differences were noted in Low-Density Granulocytes, a relative increase in the CD16^-^ compartment was observed in ISR (p=0.004). An unsupervised cluster analysis revealed the presence of three profiles with different clinical severity, unrelated to stent types or traditional risk factors.

**Conclusion:**

ISR is linked to excessive skin healing and profound alterations in cellular populations related to vascular repair and endothelial damage. Distinct cellular profiles can be distinguished within ISR, suggesting that different alterations may uncover different ISR clinical phenotypes.

## Introduction

In-stent restenosis (ISR) and stent thrombosis represent the main challenges in percutaneous coronary intervention (PCI) (1). ISR results from neointimal hyperplasia (2, 3) or from a delayed process of neoatherosclerosis. Although the development of drug-eluting stents (DES) has dramatically changed the incidence of ISR, there is still a significant proportion of patients developing ISR and requiring repeated revascularization (4, 5). Despite stent-, patient- and procedure-related factors have been postulated to explain the ISR risk, these fail to solely account for the risk of ISR and cannot fully predict ISR occurrence, thus suggesting the participation of additional factors (6).

Functional similarities between ISR and other biological processes, such as skin healing, have been proposed (7). Both ISR and hypertrophic/proliferative scars may be considered as excessive responses against an initial lesion, hallmarked by fibroblast and smooth muscle cell overactivation and proliferation (8). Although the participation of shared mechanisms in ISR and skin healing has been speculated, how this crosstalk is orchestrated is ill-defined. Several authors have postulated that inflammation may play a role in this scenario (9, 10), as it has been associated to excessive skin healing as well as to an enhanced neointimal proliferation at the coronary level. However, the exact mediators under the umbrella term of ‘inflammation’ are yet to be identified. Recent data have shed new light into the pathophysiology of the ISR from lipidomics and proteomics approaches (11, 12), although the cellular immune compartment has been largely unexplored.

Recent advances in the field of atherosclerosis and immune-mediated diseases led to the identification of novel immune populations related to the maintenance of vascular homeostasis. On the one hand, endothelial progenitor cells (EPC) have been described to migrate from the bone marrow pool in response to endothelia injury (13, 14). Similarly, angiogenic T-cells (Tang) are known to carry out vascular repair and neovascularization in adults (15). On the other hand, immunosenescence has been linked to increased endothelial damage (16). Furthermore, monocyte (17) and low-density granulocytes (LDG) (18–20) have been related to a number of vascular outcomes. However, within myeloid populations, an enormous heterogeneity has been described (21–23), including both inflammatory and anti-inflammatory or regulatory populations. Unfortunately, no studies have focused on immunosenescence or myeloid populations in the context of ISR, and only very limited evidence on EPC is available, whereas other reparative populations remain unexplored.

The identification of mediators underlying ISR may reveal potential therapeutic targets as well as novel tools (biological or inflammation-related) to explain and predict ISR occurrence, thus improving patient risk stratification and management. Taking into account the similarities between the neo-atherosclerotic pattern observed in ISR and the skin healing and atherosclerosis (24, 25), it may be hypothesized that ISR could be associated with aberrant skin healing as well as with altered levels of cell populations related to vascular homeostasis. The main aims of the present study are (i) to evaluate the association between ISR and skin healing outcomes and (ii) to evaluate whether ISR may be associated to immune cell subsets (either individual or cell signatures) related to vascular homeostasis.

## Materials and methods

### Study participants

This study was performed in the framework of the *Restenosis in Coronary Stents And Cutaneous HEaLing* [RACHEL] trial (ClinicalTrials.gov registered with accession number NCT04915391). Coronary angiographies performed in our catheterization laboratory (Hospital Universitario de Cabueñes) from 2011 to 2017 were reviewed in order to identify patients who had undergone previous stent implantation and had a catheterization date ≥ 6 months after the index procedure. Most of the angiographies (92%) were performed due to clinical criteria, while the remaining were performed as part of the routine angiographic follow-up of multicenter RCTs running in our institution. Patients were entered in the study according to the following inclusion and exclusion criteria. Inclusion criteria were (i) an age between 18 and 75 years old, (ii) having a prior stent implantation and underwent an angiogram ≥ 6 months after the index procedure, and (iii) having ISR according to Mehran classification (for the ISR group), or absence of ISR (for the ISR-free group). Life expectancy lower than one year, chronic immunomodulatory treatments (including corticosteroids), previous or current history of malignancy or inflammatory conditions, or recent infections or surgery were exclusion criteria (see registered protocol at ClinicalTrials.gov). Then, patients fulfilling with ISR type II-III or IV Mehran classification were considered as having ISR, whereas those without ISR in the second angiogram were considered as ISR-free (both ISR and ISR-free groups were confirmed by a second angiogram).

Clinical data of the patient’s profile at baseline, the characteristics of the initial PCI, including the type (BMS or DES), length and diameter of the stent and the angiographic variables of the follow-up angiography, were recorded from the database of the catheterization laboratory or from the patient’s medical records. Events at follow-up (after PCI) were also retrieved from clinical records.

Fasting blood samples were obtained from all study participants by venipuncture. Automated serum biochemical parameters, lipid analysis and complete blood counts were performed on all the participants at the Department of laboratory medicine (Hospital Universitario Central de Asturias) by means of routine laboratory methods.

### Skin biopsies and analyses

**Figure 1:**
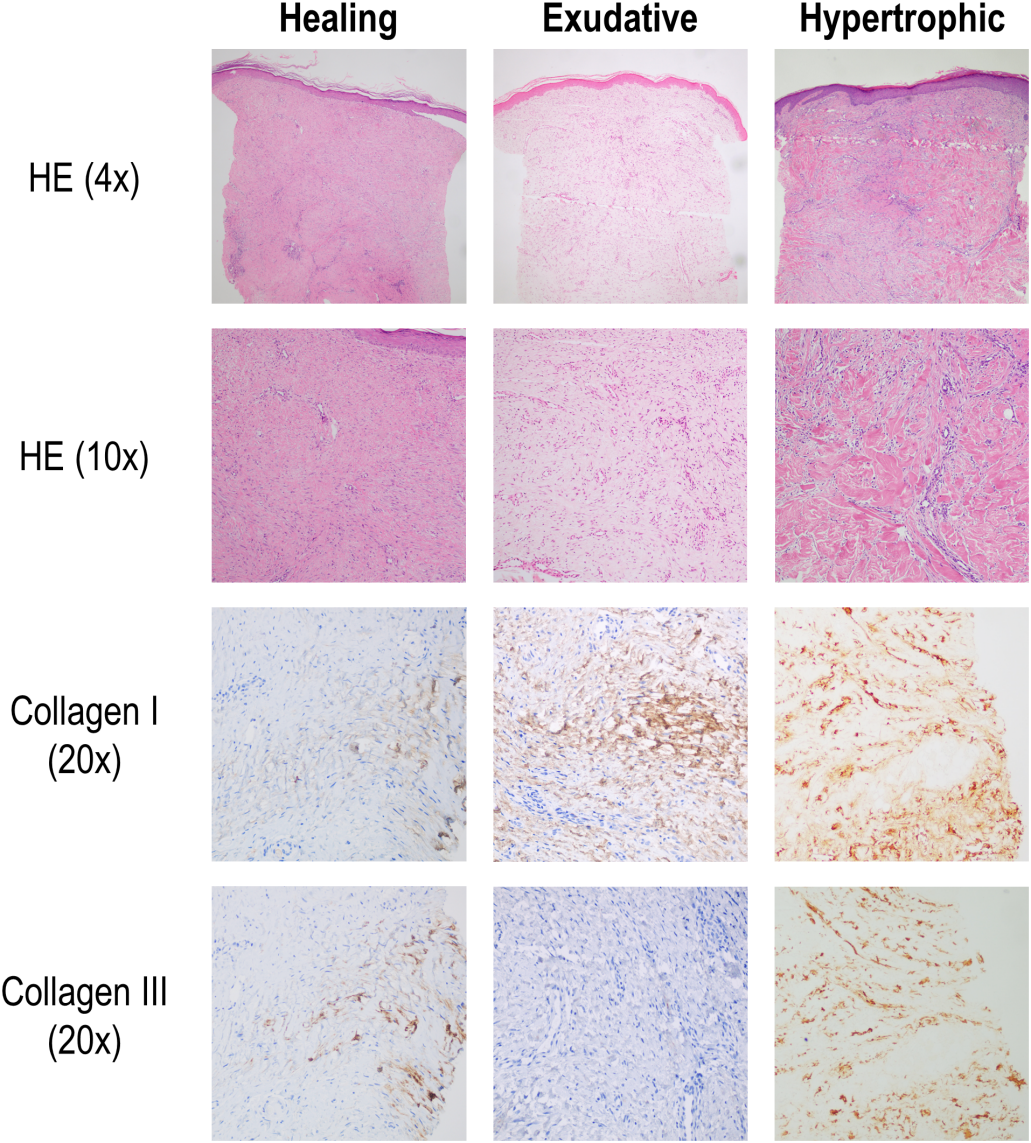
Histological analysis of skin healing patterns. The occurrence of healing patterns (healing, exudative or hypertrophic) was assessed in a second biopsy by performing skin histological preparations with Hematoxylin-Eosin staining and immunohistochemistry (IHQ) for collagen I and collagen III fibers. Healing pattern was hallmarked by myofibroblasts/fibroblast with horizontal orientation relative to epidermis and higher abundance of collagen III over collagen I. Exudative pattern was defined as showing vertical orientation of myofibroblasts/fibroblasts, with a collagen I predomination over collagen III. Hypertrophic pattern was characterized by having coarse collagen, bundle-like deposition, with higher collagen I over collagen III expression. Representative images of each pattern per staining are showed.

A 6-mm-diameter baseline biopsy was performed on the shoulder on healthy skin after the blood sample was taken. The second biopsy (4 mm of diameter), object of analysis of the study, was obtained at 6 weeks by the same procedure and sent to the Department of Pathology Anatomy for an assessment of the healing outcomes (26). Biopsies were analyzed in a microscope Olympus BX43 (Olympus, Germany) equipped with a camera Olympus DP74. Images were acquired under the software Olympus cellSens Entry version 1.7. Under normal conditions, after 6 weeks, the scar produced by the first biopsy should be in a healing phase in which, by hematoxylin-eosin staining, myofibroblasts/fibroblasts appear in a horizontal orientation in relation to the epidermis and, using immunohistochemical techniques, it should be positive for vimentin, with smooth muscle actin and desmin being negative. Furthermore, in this phase the synthesized collagen should be predominantly type III. Alternatively, if a perpendicular orientation of the myofibroblasts/fibroblasts as well as positivity for smooth muscle actin and/or predominance of type I collagen were observed, the healing outcome would be classified as the exudative-productive phase, which would entail a delay in healing. Pathological/proliferative scarring in the form of a hypertrophic scar, with a swirling orientation of the collagen fibers and the presence of coarse collagen bundles, was also assessed (27, 28). Therefore, differences across the three healing patterns (healing, exudative or hypertrophic) or depending on the presence of proliferative scars (hypertrophic vs. healing+exudative) were analyzed (**Figure 1**).

### Analysis of cellular populations

Blood samples were immediately transported to the laboratory and processed. For all flow cytometry analyses, specific compensations and panel design were implemented according to good laboratory practice. EPC were analyzed by flow cytometry following EUSTAR recommendations (29) with few modifications as previously described (30). After preincubation with FcR blocking reagent (Miltenyi Biotech) to prevent unspecific antibody binding, whole blood was stained with anti-CD34-FITC (BD Pharmigen, Germany), anti-VEGFR2-PE (R&D, Germany) and anti-CD133-APC (Miltenyi Biotech, Germany) or identical isotype antibodies (BD Pharmigen) for 30 minutes at 4°C. Red blood cells were then lysed and washed. After gating the lymphocyte population, CD34-positive events were selected and CD34/VEGFR2/CD133 triple-positive were considered as EPC. VEGFR2-positive events within the lymphocyte gate and lacking CD34/CD133 expression were considered as detached, mature endothelial cells (EC) (30).

In parallel, peripheral blood mononuclear cells (PBMCs) were obtained by centrifugation on density gradient (18, 31). Then, PBMCs were incubated with CD14-FITC (Immunostep, Spain), CD16-APC-Cy7 (BioLegend, Germany) and ACE-APC (Miltenyi Biotech); or CD3-PerCP-Cy-5,5 (Tonbo Biosciences, Belgium), CD184-PE-Cy7 (BD Biosciences, Germany), CD31-FITC (BD Biosciences), CD4-PE (Immunostep) and CD28 APC-Cy7 (Thermo Fisher, Germany); or CD14-FITC (Immunostep), CD15-PE-Cy7 (Miltenyi Biotech), CD16-APC-Cy7 (BioLegend); or corresponding isotype antibodies for 30 minutes at 4°C protected from light. Next, cells were washed twice with PBS and analyzed by flow cytometry (FACS Canto II (BD Biosciences) with FACS Diva 6.5 software).

Then, a ‘live gate’ excluding debris and subcellular events was designed. Lymphocytes, monocytes and granulocyte regions were defined according to their FSC/SSC features and gating strategies were follow as previously described by our group (18, 31) for the identification of Tang (CD3^+^CD31^+^CD184^+^), CD4^+^Tang and CD8^+^Tang subpopulations, senescent T-cells (CD4^+^CD28^null^), monocyte subsets (classical (CD14^+^CD16^-^), intermediate (CD14^+^CD16^+^) and non-classical (CD14^low^CD16^+^) monocytes), ACE expression, total LDG (CD15^+^LDG) and LDG subsets (CD14^-^CD16^-^CD15^+^ and CD14^low^CD16^+^CD15^+^). The frequency of each population was referred to the parental gates unless otherwise stated.

### Statistical analyses

Continuous variables were expressed as median (interquartile range) or mean ± standard deviation, according to the distribution of the variables. Categorical variables were summarized as n (%). Differences between groups were assessed by Mann Whitney U, Kruskal-Wallis or chi-squared tests, as appropriate. Logistic regression models, either univariate or multivariate adjusted by confounders, were used to evaluate the association between ISR and skin healing outcomes. Odds ratios (OR) and 95% confidence intervals (CI) were computed. Unsupervised cluster analysis was performed with variables showing associations at p<0.100 using squared euclidean distances and Ward’s Minimum Variance Method, in order to identify clusters minimizing the loss of information. R package heatmap.2 was used to generate the heatmap for visualization purposes. A p-value<0.050 was considered as statistically significant. Statistical analyses were performed with SPSS 27.0 and GraphPad Prism 8.0 for Windows.

### Ethics statement

Approval for the study was obtained from the Institutional Review Board (Comité de Ética de la Investigación con Medicamentos del Principado de Asturias, reference 90/16), in compliance with the Declaration of Helsinki. All participants gave a written informed consent prior to their inclusion in the study.

## Results

### ISR is associated with excessive skin healing response

Coronary angiograms (n=9236) were reviewed and a total of 285 patients were found to meet inclusion criteria, from which 60 were selected and divided into (i) a group of cases exhibiting ≥1 previous stent implantation (15 with BMS and 15 with DES) with ISR confirmed in a second angiogram (presenting with clear type II-IV restenosis grades, and absence of diffuse disease), and (ii) a control group made up of 30 patients with ≥1 previous BMS stent implantation who had not suffered ISR at follow-up (ISR-free) (selected among those with a total absence of intrastent hyperplasia) (**Table 1**). All patients had confirmed their ISR or ISR-free status in a second angiogram. These groups did not differ in the frequency of traditional risk factors (hypertension: p=0.426, diabetes: p=0.273, dyslipidemia, p=0.278, and smoking p=0.271). Time between the index interventional procedure and the angiographic follow-up was 30 (91) months and 39 (46) months between the angiographic follow-up and the blood sample. The interval between the index procedure and the blood sample was 87 (range 3 - 220) months.

**Table 1:** Description of study population.

	All patients	ISR	
		No	Yes
n	60	30	30
Demographics			
Age (years), mean ± SD	60.10 ± 7.02	60.86 ± 6.20	59.37 ± 7.74
Sex, n men/women	51/9	25/5	26/4
Clinical features			
Diseased vessels (number), mean ± SD	1.88 ± 0.80	1.77 ± 0.77	2.00 ± 0.83
Type of stent, n BMS/DE	45/15	30/0	15/15
Stent length (mm), mean ± SD	22.65 ± 15.17	19.77 ± 8.07	25.53 ± 19.65
Diameter (mm), mean ± SD	3.33 ± 0.37	3.43 ± 0.31	3.24 ± 0.41
Stable angina, n(%)	26 (43.3)	16 (53.3)	10 (33.3)
Events at follow-up, n(%)	7 (11.6)	0 (0.0)	7 (23.3)
Diabetes, n(%)	20 (66.6)	8 (26.6)	12 (40.0)
Dyslipidemia, n(%)	51 (85.0)	24 (80.0)	27 (90.0)
Smoking, n(%)	54 (90.0)	28 (93.3)	26 (86.6)
Hypertension, n(%)	37 (61.6)	20 (66.7)	17 (56.7)
Skin healing outcomes			
Patterns, n He/Ex/Hy	29/15/16	14/11/5	15/4/11
Hypertrophic outcome, n(%)	16 (26.6)	5 (16.6)	11 (36.6)
Blood cell counts			
Leukocytes, mean ± SD	7.63 ± 2.54	7.10 ± 2.43	8.11 ± 2.54
Lymphocytes, mean ± SD	2.23 ± 0.82	2.05 ± 0.69	2.40 ± 0.89
Monocytes, mean ± SD	0.53 ± 0.20	0.48 ± 0.16	0.58 ± 0.22
Neutrophils, mean ± SD	4.43 ± 1.94	4.14 ± 1.75	4.52 ± 2.21

Demographics and clinical features of study participants, as whole group and stratified according to ISR status. Variables were summarized as mean ± SD or n(%), as appropriate. He, healing; Ex, exudative; Hy, hypertrophic.

After 6 weeks, study participants undergone a second biopsy, and pattern analyses (**Figure 1**) revealed that normal healing pattern was found in 29 (48.3%) individuals, whereas hypertrophic and exudative patterns were observed in 16 (26.6%) and 15 (25.0%) participants, respectively. Hypertrophic outcome was more frequent among patients with ISR compared to those ISR-free (11(36.6%) vs 5(16.6%)), although statistical significance was not reached (p=0.077). However, multivariate analysis revealed that skin hypertrophic pattern was an independent predictor of ISR occurrence after adjusting for clinical features and risk factors (diabetes, smoking, hypertension, dyslipidemia, stent length and stent diameter) (**Table 2**). Equivalent results were obtained in an extended cohort (n=80) as per our initial protocol (data not shown).

**Table 2:** Association between ISR and excessive skin healing (hypertrophic pattern).

	OR	95% CI	p-value
Univariate			
Hypertrophic pattern, yes	2.895	0.860 – 9.745	0.077
Multivariate			
Hypertrophic pattern, yes	4.544	1.044 – 18.073	**0.033**
Stent length (mm), per unit	1.047	0.996 – 1.100	0.074
Diameter (mm), per unit	0.151	0.024 – 0.956	**0.045**
Diabetes, yes	1.630	0.420 – 6.322	0.480
Dyslipemia, yes	3.376	0.452 – 25.178	0.174
Smoking, yes	0.239	0.030 – 1.886	0.174
Hypertension, yes	0.460	0.119 – 1.779	0.260

The association between ISR and hypertrophic pattern occurrence was analyzed by logistic regression in univariate and multivariate models. P-values reaching statistical significance were highlighted in bold.

These results reinforce the independent association between ISR and skin healing patterns, patients with ISR being more likely to exhibit hypertrophic skin outcomes.

### ISR is associated with altered levels of several cell populations linked to endothelial damage and vascular repair

**Figure 2:**
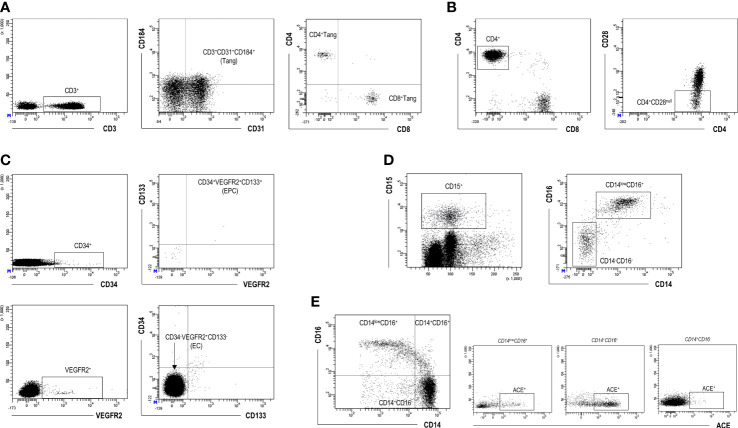
Flow cytometry analysis of cellular populations. Gating strategy for the identification and quantification of Tang **(A)**, CD4^+^CD28^null^ **(B)**, EPC and EC **(C)**, LDG **(D)**, and monocyte subsets **(E)** by flow cytometry. Dot-plots from a representative patient are shown.

Next, the blood levels of a number of cellular populations involved in endothelial damage and vascular repair processes were evaluated by flow cytometry (**Figure 2**).

**Figure 3:**
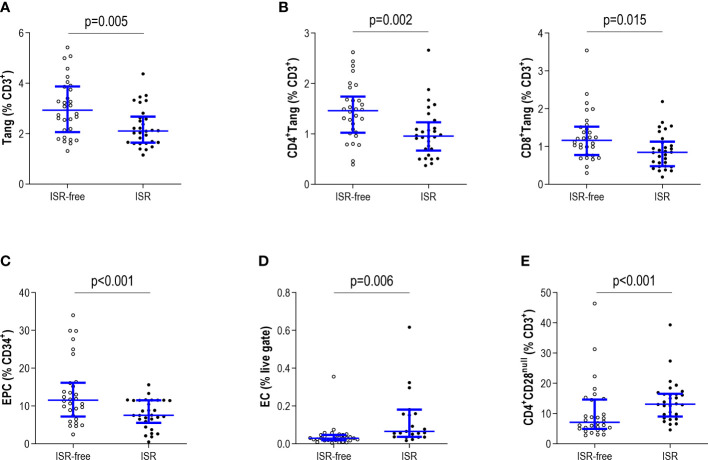
Analysis of circulating endothelial cell subsets and T-cell subpopulations in ISR. The Tang **(A)**, CD4^+^ and CD8^+^ Tang subsets **(B)**, EPC **(C)**, EC **(D)** and CD4^+^CD28^null^ **(E)** subsets were evaluated by flow cytometry and levels were compared between patients with ISR (black dots) and ISR-free patients (open dots). Each dot represents one individual. Upper and lower bars represent 75^th^ and 25^th^ percentiles and medium bars correspond to the median values. Differences between groups were assessed by Mann-Withney U tests.

Patients with ISR exhibited lower circulating levels of Tang and EPC populations compared to those without ISR (**Figures 3A–C**). No differences in the frequency of CD4 (ISR: 46.81(14.19)% vs ISR-free: 46.95(18.66)%, p=0.595) or CD8 (41.46(11.92) vs 38.22(11.62)%, respectively, p=0.228) usage within the Tang pool were observed between groups. Then, both CD4^+^Tang and CD8^+^Tang subsets were reduced to the same extent in ISR (**Figure 3B**). On the contrary, EC counts were found to be increased linked to ISR (**Figure 3D**). Similarly, senescent T-cells (CD4^+^CD28^null^) were shown to be elevated in patients with ISR compared to their ISR-free counterparts (**Figure 3E**). Of note, no differences in the total number of leukocytes (p=0.074) or lymphocytes (p=0.196) were observed between groups. Consequently, equivalent results were observed when absolute levels of those populations were computed (Tang: p=0.015; EPC: p=0.029; EC: p=0.009; and CD4^+^CD28^null^: p=0.003).

**Figure 4:**
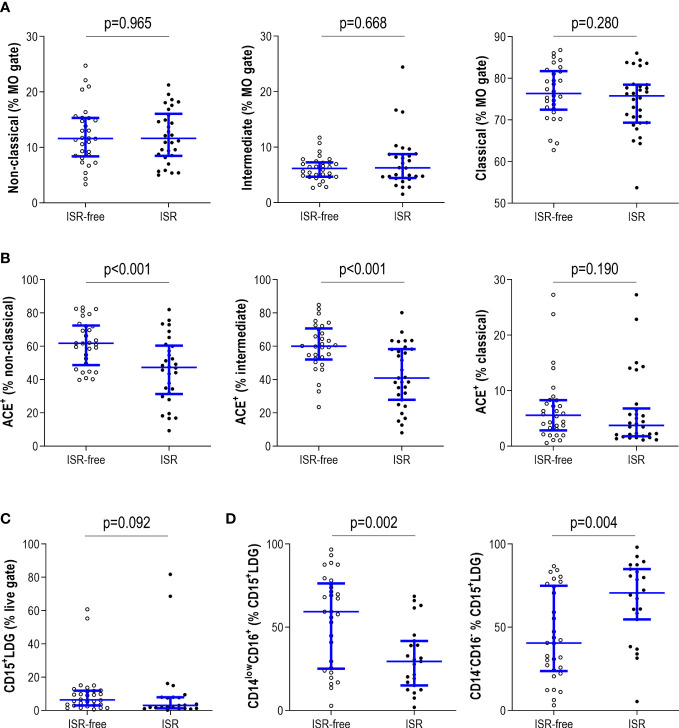
Analysis of myeloid subsets in ISR. The monocyte subsets (non-classical, intermediate and classical) were identified by means of their CD14/CD16 expression. Their frequency **(A)** and ACE expression **(B)** were compared between patients with ISR (black dots) and ISR-free patients (open dots). **(C)** The circulating levels of total LDG and **(D)** their subtypes based on CD14/CD16 expression were also compared between patients with ISR (black dots) and ISR-free patients (open dots). Each dot represents one individual. Upper and lower bars represent 75^th^ and 25^th^ percentiles and medium bars correspond to the median values. Differences between groups were assessed by Mann-Withney U tests.

Next, whether differences were observed within the monocyte pool was evaluated. No differences were observed in the frequency of monocyte subsets according to ISR status (**Figure 4A**). However, the proportion of ACE-expressing non-classical and intermediate monocytes was higher in ISR-free patients compared with their ISR counterparts, although no differences were found in the classical subset (**Figure 4B**). No differences were noticed in the total counts of monocytes between both groups (p=0.091). Equivalent results were obtained when absolute counts were computed for monocyte subsets (all p>>0.050).

Finally, although no differences were noted in the number of total LDG (CD15^+^LDG) (**Figure 4C**), diverging patterns were retrieved when the LDG subsets were analyzed. ISR was associated with an expansion of the CD14^-^CD16^-^CD15^+^ compartment at the expense of a reduction in that of CD14^low^CD16^+^CD15^+^ (**Figure 4D**). These differences were maintained when evaluated within the total live gate region (CD14^-^CD16^-^CD15^+^: p=0.010; CD14^low^CD16^+^CD15^+^: p=0.009).

Of note, no changes were observed for any cell population when compared on the occurrence of skin hypertrophic scars (**Supplementary Table 1**), hence strengthening the notion that the alterations observed were ISR-driven. Moreover, none of these populations were related to the intervals between the index procedure and the second catheterization or the recruitment (**Supplementary Table 2**). The fact that the ISR-free group was enriched in patients with BMS suggest that those findings were related to genuine mechanisms of ISR protection (as BMS are more likely to lead to ISR), without potential external confounders being involved. Finally, within the ISR group, no differences in the frequency of any cell population were observed in relation to stent types (BMS vs DES) (all p>0.050, data not shown).

Taken together, all these findings suggest that ISR was associated with specific alterations in cellular populations indicative of impaired vascular repair and enhanced endothelial damage. These changes were observed in the lymphoid and myeloid lineages, thus suggesting a broad involvement of the systemic cellular compartment in the setting of ISR.

### Distinct cellular clusters with clinical relevance can be distinguished within ISR

In addition to evaluate univariate differences across ISR status, we aimed to evaluate whether patterns of differences can be detected in association with clinical features within ISR. Then, populations found to exhibit associations with ISR were entered into an unsupervised cluster analysis.

**Figure 5:**
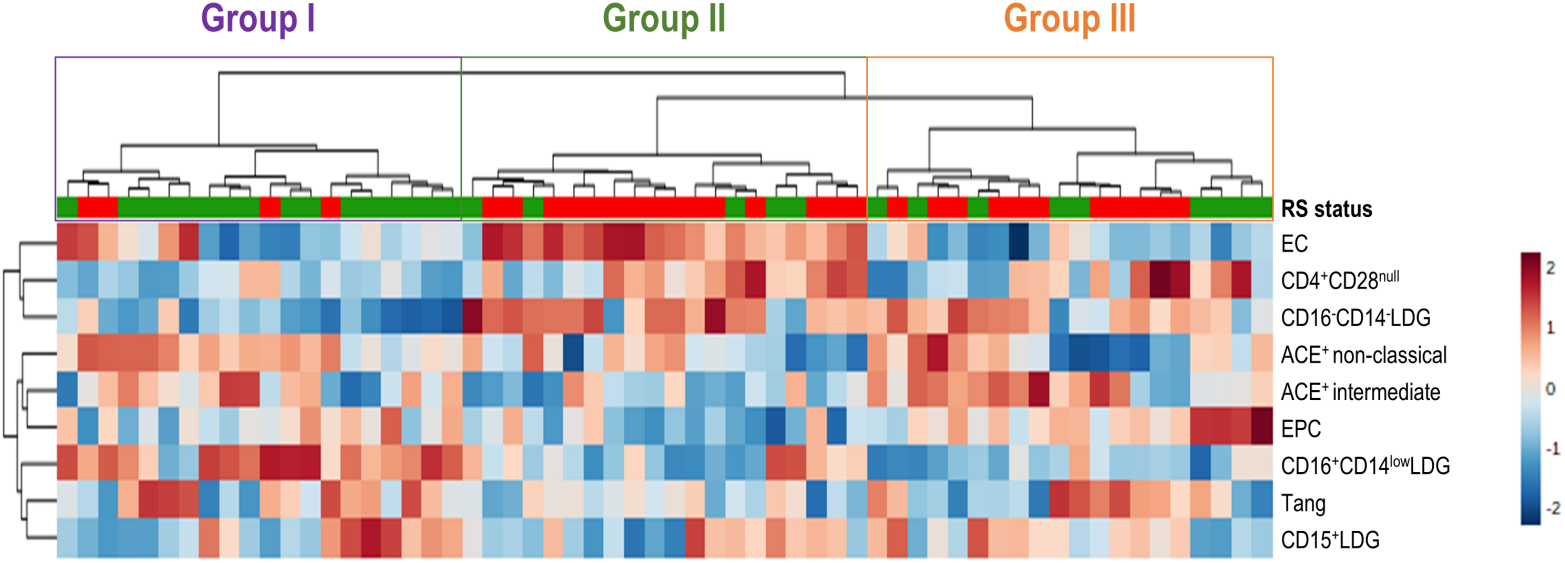
Cluster analysis of cellular subsets. Heatmap showing the dendrogram classification of the clusters based on the cellular populations exhibiting alterations in ISR. Upper bar denotes RS status (green: ISR-free, red: ISR). Left dendogram showed the grouping patterns of the variables. Each column represents an individual, and each row represents one variable. The three clusters identified are indicated with colored rectangles over the dendogram (top). Tiles are colored based on frequencies, red and blue indicating low or high levels, respectively (see legend at the right).

A total of three groups were detected in our analysis (**Figure 5**): group I (hallmarked by the relative highest level of vascular repair populations, low immunosenescence, high ACE expression and a CD16^+^-shifted LDG pool), group II (characterized by profound alterations in vascular repair populations and endothelial damage, high immunosenescence and decreased ACE expression on monocytes) and group III (hallmarked by a strong decrease in Tang but mild alterations in EPC and LDG profile, enhanced immunosenescence, and high ACE expression) (**Supplementary Figure 1**). Furthermore, these groups underlie differences at the clinical level. Patients using group II were found to exhibit a higher number of diseased vessels and implanted stents, in addition to higher rates of ISR and previous PCI as well as more likely to develop events at follow up, compared to groups I and III (**Table 3**). A non-significant, slightly higher occurrence of proliferative skin healing was noted, whereas a distinct distribution of skin healing patterns was detected. Furthermore, group III was found to be associated with a different, less severe clinical vascular presentation. Therefore, group II could be related to a more severe clinical risk profile, despite no differences in demographics, traditional cardiovascular risk factors and type of stent.

**Table 3:** Association between clusters and clinical features.

	Group I	Group II	Group III	p-value
n	20	20	20	
Demographics				
Age (years), mean ± SD	62.80 ± 4.83	57.95 ± 7.40	59.75 ± 7.74	0.089
Sex, n men/women	4/16	1/19	4/16	0.308
Clinical features				
Diseased vessels	1.76 ± 0.75	2.30 ± 0.51	1.69 ± 0.74	**0.012**
Number of stents	1.25 ± 0.82	2.50 ± 1.02	1.80 ± 0.78	**0.010**
Type of stent, n BMS/DES	17/3	12/8	16/4	0.155
Stent length, mean ± SD	18.05 ± 8.19	26.55 ± 17.61	24.35 ± 17.33	0.112
Diameter, mean ± SD	3.37 ± 0.35	3.23 ± 0.39	3.40 ± 0.38	0.430
ISR, n(%)	4 (20.0)	15 (75.0)	11 (55.5)	**0.002**
Stable angina, n(%)	3 (15.0)	10 (50.0)	14 (70.0)	**0.002**
Events at follow-up, n(%)	0 (0.0)	6 (30.0)	1 (5.0)	**0.007**
Hypertension, n(%)	15 (75.0)	9 (45.0)	13 (65.0)	0.139
Diabetes, n(%)	7 (35.0)	5 (25.0)	8 (40.0)	0.592
Dyslipidemia, n(%)	17 (85.0)	18 (90.0)	16 (80.0)	0.676
Smoking, n(%)	6 (30.0)	v9 (45.0)	4 (20.0)	0.231
Skin healing outcomes				
Patterns, n He/Ex/Hy	9/11/3	8/3/7	12/1/6	**0.029**
Hypertrophic outcome, n(%)	3 (15.0)	6 (35.0)	6 (30.0)	0.126

Differences across groups were evaluated by Kruskal-Wallis or chi-squared tests, as appropriate. P-values reaching statistical significance were highlighted in bold. He, healing; Ex, exudative; Hy, hypertrophic.

Altogether, these results suggest that differences in circulating cellular populations may inform distinct profiles within ISR. Hence, different alterations at the cellular level may uncover different ISR phenotypes at the clinical level, in terms of clinical severity, risk and extension, independently of traditional risk factors and stent types.

## Discussion

**Figure 6:**
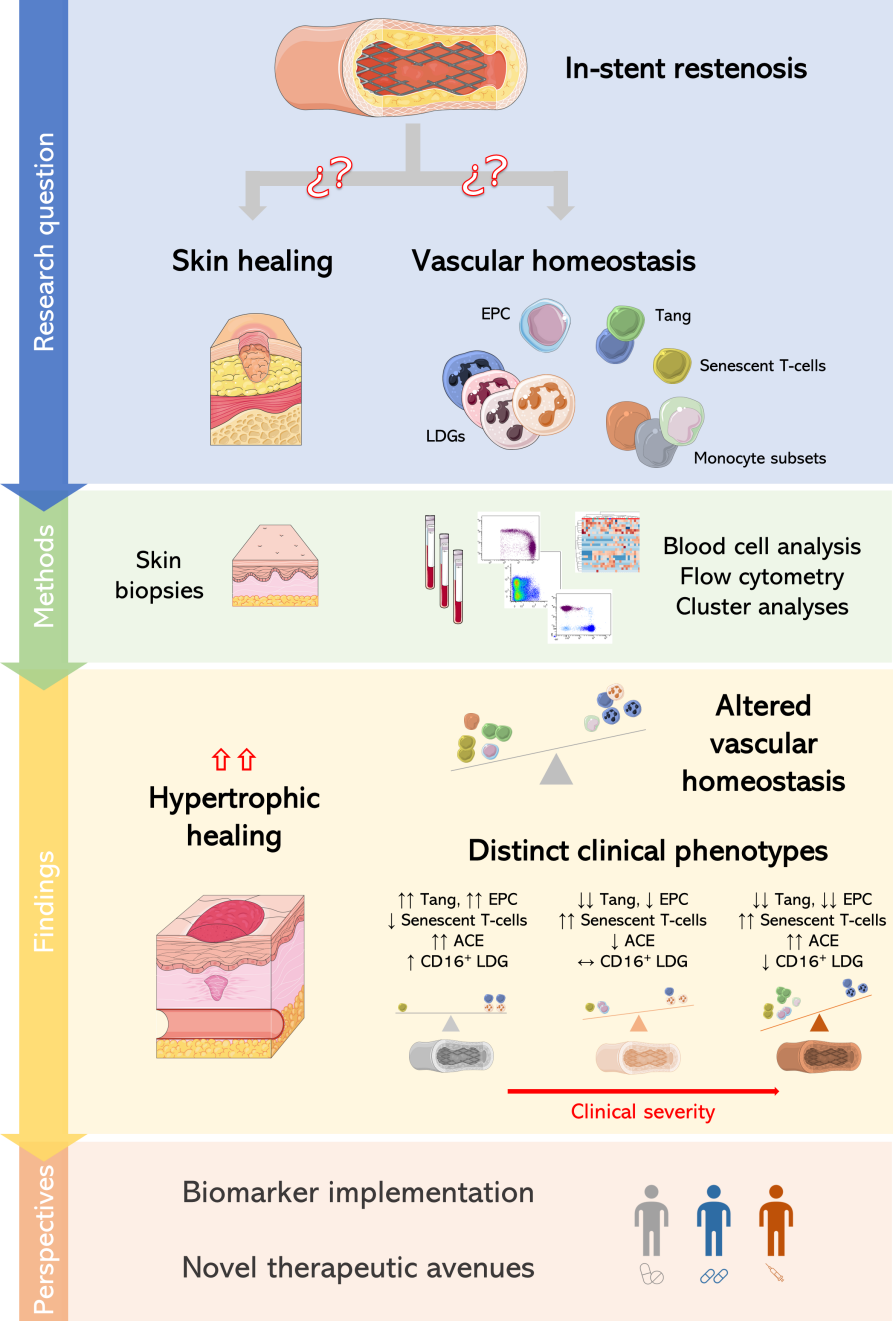
Global overview of the present study. Project phases and results obtained in this study are depicted.

The global burden of ISR remains a significant challenge for human health in terms of poor clinical outcomes, quality of life, and medical costs and services. Despite the latest advances on stent technology and management, there is still significant room for improvement (6). Gaining understanding towards ISR etiopathogenesis, especially at the cellular level, may shed new light into novel clinical procedures and disease targets to guide personalized medicine approaches. The results herein reported align two-fold with this goal. First, our results point to a connection between ISR and excessive skin healing outcomes. Moreover, ISR was found to be related to profound alterations in several cellular mediators of vascular homeostasis. These alterations informed different clinically-relevant clusters of patients (**Figure 6**). To the best of our knowledge, this is the first study to present a comprehensive characterization of cellular subsets in ISR, including the assessment of the Tang population in this scenario, as well as to prospectively evaluate the association between ISR and skin healing.

Our findings uncover an association between ISR and skin healing. A hypertrophic outcome upon provoked skin healing was found to be a significant predictor for ISR status. In line with our results, Odzol and coworkers had found a higher prevalence of restenosis among patients with proliferative scars upon previous open-heart surgery (7). Interestingly, stent length, diabetes and proliferative scars were independent predictors of ISR occurrence in their retrospective analysis (7). This association may suggest that both ISR and skin healing are functionally connected, probably due, at least in part, by shared mechanisms. The results of our study offer validation to the connection between both processes independently of traditional risk factors in a prospective design after provoking skin healing in a selected, matched groups of patients. Moreover, it also provides a step forward in this field by shedding new light into the role of inflammation and immune circuits which may be involved in both excessive responses.

Although systemic inflammation and immunity have been described to contribute to cardiovascular outcomes in several contexts, from basic studies to large clinical trials (such as CANTOS or LoDoCo2) (32, 33), the exact links in the setting of ISR remain poorly characterized. Moreover, current inflammatory biomarkers are limited by their lack of specificity and poor reflection of the underlying processes (34–37). Our results suggest that multiple cell populations, belonging to different lineages, show alterations in ISR. This finding aligns with the complexity of ISR by suggesting a multifaceted pathogenesis at the cellular level. One of the most remarkable results of our work was the analysis of cellular populations involved in vascular repair. EPC had been described to be altered in frequency and/or functionally impaired in ISR patients (38), although results have been controversial (39, 40), probably due to technical limitations, misleading phenotypical characterization and lack of assay harmonization (40). Our results, using specific international validated guidelines for EPC quantification, support a strong EPC depletion in ISR, in accordance with other vascular disorders [reviewed in (41)]. On the other hand, our findings revealed a similar picture for the Tang subset in this scenario. Importantly, an equivalent reduction in CD4+ and CD8+ subsets was observed, hence pointing to a global effect on the Tang population rather than on their specific compartments. This is in line with previous results in other, non-autoimmune conditions (18). Tang cells are known to participate in adult vasculogenesis and vascular repair, and Tang depletion has been linked to cardiovascular outcomes in several immune-mediated and chronic conditions (42–47). However, their role in ISR had not been explored. Therefore, our findings reinforce the notion that reparative mechanisms are profoundly impaired in ISR and expand the knowledge about the role of Tang in vascular homeostasis to a broader range of human diseases. Functional studies to evaluate the impact of Tang activity on the vascular repair in the setting of ISR, as well as their cooperative effects with other cellular subsets involved in vascular homeostasis are warranted.

Furthermore, these results may be of application for the clinical setting. Although improvements in stent technology have led a dramatic decrease of ISR occurrence, a plateau seems to have been reached. Our findings may open a new therapeutic avenue to act on inflammation at the lesion level through stent re-formulations. EPC-capturing stents have been largely proposed to reduce ISR burden by neointimal hyperplasia inhibition (48). However, trial results have been suboptimal, and potential limitations to efficiently capture EPC have been proposed (49–51). Moreover, an insufficient autologous EPC pool, impaired mobilization and/or the need of *in vitro* expansion for subsequent EPC infusions add important layers of complexity to this intervention. Therefore, the use of dual EPC- and Tang-capturing stents may represent a potential alternative. Higher frequency of Tang, better phenotypical characterization and established knowledge on T-cell proliferation and expansion protocols may be important advantages in this regard.

On the contrary, other cell subsets such as EC and senescent T-cells were found to be increased in ISR. CD4^+^CD28^null^ expansions have been related to severe clinical cardiovascular outcomes (52), but their involvement in ISR was unexplored. These results underline the potential contribution of T-cell exhaustion in ISR, thus warrantying an analysis of immune-senescence and inflammaging in this context from a basic perspective. Whether rejuvenating, counteracting interventions may be beneficial in ISR remains plausible. Concerning the myeloid compartment, although no major differences in monocyte subsets were retrieved, reduced ACE expression was associated with ISR. This may be in line with the lack of positive effects of ACE inhibitors on neointimal progression (53), thus suggesting that certain ACE activity (or some isoforms, or ectopic expression) may be protective in ISR. Also within the myeloid lineage, although no major differences were observed in the total pool of LDG populations, qualitative alterations were found. Interestingly, a CD16^-^LDG shifted profile was linked to ISR, in line with other chronic conditions (18). These cell mediators have been demonstrated to exhibit a more immature phenotype, probably related to an excessive stimulation of bone marrow reservoir, also linked to vascular outcomes (54–56). A selective enrichment of CD16^+^LDG was observed in the ISR-free group, which may suggest a protective effect of this population. In fact, suppressive or regulatory functions have been described for this cell subset in a number of contexts (23, 57). Taken together, these findings add to the functional and phenotypical heterogeneity of LDG in human disease and support the idea that the myeloid pool is more complex than initially considered. Importantly, our findings overall strengthen the notion that vascular outcomes should not be regarded as the consequence of a passive accumulation of endothelial damage stimuli, as reparative mechanisms were demonstrated to be impaired as well. Therefore, the results herein reported reinforce the need of a paradigm change towards an (im) balance of vascular homeostasis, with both damage and reparative processes being implicated.

A strong point from our research was the integrative approach, which had not been explored in ISR until date. Single biomarkers or mediators are unlikely to inform on complex scenarios and, unavoidably, always provide limited knowledge. A fundamental critique to the contemporary literature in ISR is that the focus is made on individual cell populations or biomarkers, thus lacking information on the context and the global picture of interactions and cellular networks. However, in a complex scenario such as ISR, this conventional approach is suboptimal and overly simplistic. Our study has pioneered the use of an unsupervised clustering approach together with a comprehensive assessment of cell populations involved in vascular homeostasis, hence providing a global, integrative picture, overcoming the limitation of lacking mechanistical insights, and adding incremental value over conventional clinical features. Our approach revealed that although differences in individual mediators occur in ISR, not all are present in the same individuals, neither to the same extent, and certain associations can be found among particular cell mediators. As a consequence, groups of cellular traits can define clusters of patients with different clinical characteristics, that is, different clinical ISR phenotypes. Our approach revealed the existence of three cellular clusters that cover the whole spectrum of vascular homeostasis status: from a complete impairment of repair mechanisms, qualitatively altered monocyte subsets, high immunosenescence and a CD16^–^shifted LDG pool, indicative of central haematopoietic traits (group II); to a partial vascular repair impairment, high immunosenescene and a mildly skewed LDG profile, indicative of a partial loss of homeostasis (group III); and to a relative-normal vascular repair, with no immunosenescence and no major signs of endothelial damage (group I). Clinical features parallel this spectrum, from a high-risk situation with higher and larger lesions (II), to lower but higher number of lesions (III), and less severe lesions and risk profiles (I), respectively. Of note, these clinical phenotypes were independent of traditional risk factors, thus providing an incremental value for the clinical setting that cannot be obtained from existing clinical instruments or single candidate biomarkers. Moreover, none of cellular populations were related to time vintages from index procedure, thus suggesting that those alterations and composite clusters are rather stable along ISR course. Therefore, the observation of these clinical clusters may be of assistance to resolve clinical heterogeneity and to guide individualized care in ISR, a major unmet need in this area (6). With an increasing number of therapeutic options and pharmacological armamentarium, the identification of these profiles may be key for a targeted management. Finally, these findings allow to gain understanding towards the cellular architecture of ISR pathogenesis by identifying not only novel mediators, but also potential connections among them. Our analysis has helped us to unveil associations among groups of mediators, by relating the CD16^-^LDG expansion to endothelial damage and immunosenescence features, as previously suggested for other disorders (52), as well as showing a closer association among reparative populations, ACE expression and the CD16^+^LDG enrichment. Functional studies are warranted to deepen into these concepts.

This study has a number of limitations that must be remarked, such as the reduced sample size, the case-control design, angiographic vs causal definition of ISR, and the high variability in stent types and in the periods between the initial PCI and the control angiography, as well as between the initial PCI and patients’ entry in the study or second catheterization. Importantly, skin biopsies were obtained long after stent implantation. Although this facilitates study methodology and ensure a proper ISR classification, this retrospective design may limit the interpretation of the findings since pathogenic mechanisms occur at different time points. However, neither stent status nor those intervals were related to any of the immune cell subsets analyzed, hence ruling out a major effect of time vintages in the present study. Importantly, the groups of patients recruited are representative or real-world ISR populations, covering the whole spectrum from early to late phases upon stent implantation. Additionally, although BMS/DES head-to-head comparative studies may be needed for validation, it must be noted that the former are no longer used, which challenges the appraisal of such limitation. However, it must be noted that no effect was observed in our analysis. Furthermore, studies characterizing these populations in relation to causes of restenosis are warranted. Nevertheless, it must be noted that the latter apply to the local tissue features, whereas this study is focused on the systemic compartments, and distinct subsets/endotypes may be expected. Finally, no functional characterization of the cellular populations analyzed was performed, so mechanistic insights of this study are limited.

In conclusion, ISR was associated with excessive healing outcomes at the skin level, as well as with profound alterations in immune populations related to repair and endothelial injury at the systemic level. Distinct cellular signatures can be distinguished within ISR, thus suggesting that different cellular alterations may uncover different ISR clinical phenotypes, with clinical added value beyond traditional risk factors. This may lay the foundation for individualized approaches in the clinical setting, hence opening an innovative horizon for research and practice in interventional cardiology. Larger studies, with longer follow-up periods and simultaneous skin biopsies and stent deployment are needed to confirm these findings and provide clinical validation.

## Data availability statement

The original contributions presented in the study are included in the article/**Supplementary Material**. Further inquiries can be directed to the corresponding author.

## Ethics statement

The studies involving human participants were reviewed and approved by Comité de Ética de la Investigación con Medicamentos del Principado de Asturias. The patients/participants provided their written informed consent to participate in this study.

## Author contributions

IL and JR-C performed most of the experimental procedures and carried out the statistical analyses. IL and RB performed all the clinical procedures and were in charge of patients’ recruitment. IR, MP, RR-A, DR, ME-A, SL, and ÁM carried out some experimental procedures and analysis. All authors contributed to the data analysis interpretation and discussion of the results. IL, AS, and JR-C drafted and edited the manuscript. IL, RB, IR, ÁM, AS, and JR-C participated in the conception of the study and designed the protocols. All authors read the manuscript, revised it for intellectual content, approved the final version, and agreed to be accountable for all aspects of the work.
