# Supplementary figures and images for: In-stent restenosis is associated with proliferative skin healing and specific immune and endothelial cell profiles: results from the RACHEL trial
Source: Front Immunol. 2023 May 31;14:1138247. doi: 10.3389/fimmu.2023.1138247 (PMC10265483; doi:10.3389/fimmu.2023.1138247)

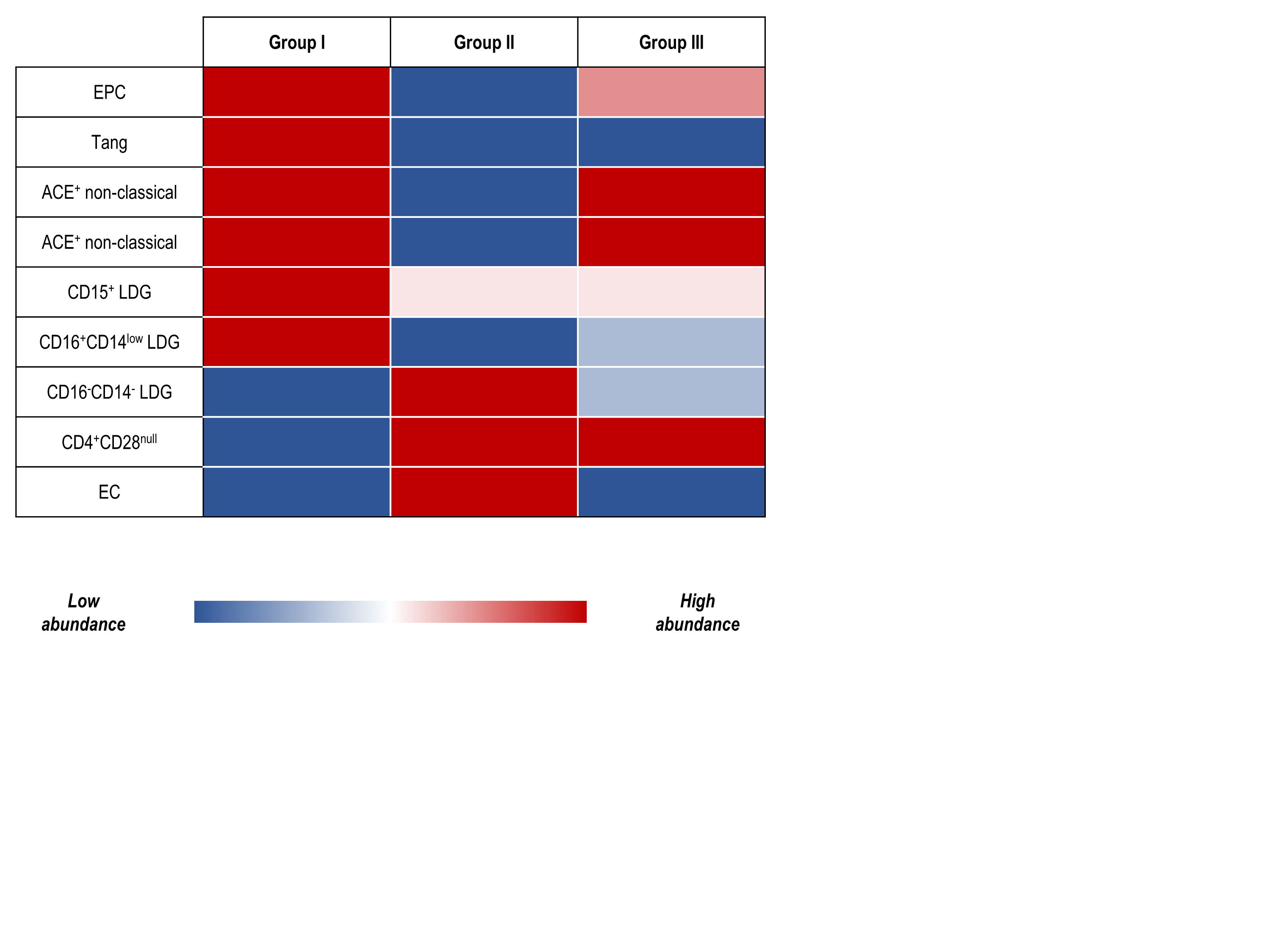

Supplement: Supplementary file 1 [file Image_1.tif]
