# Supplementary material for: In-stent restenosis is associated with proliferative skin healing and specific immune and endothelial cell profiles: results from the RACHEL trial
Source: Front Immunol. 2023 May 31;14:1138247. doi: 10.3389/fimmu.2023.1138247 (PMC10265483; doi:10.3389/fimmu.2023.1138247)
Supplement: Supplementary file 2 [file DataSheet_1.docx]

**SUPPLEMENTARY MATERIALS**

**Supplementary Figure 1: Infographic of levels of cell populations entered in cluster analysis.** The levels of cell populations entered in the cluster analysis (left, in rows) are depicted in colours according to each group resulting from the cluster analysis (in columns). The colours indicate relative abundance according to the legend (bottom), from blue (low abundance) to red (high abundance), based on median values.

**Supplementary Table 1: Association between the levels of cell populations and skin healing outcomes.** Levels were expressed as median (interquartile range) and differences were assessed by Mann-Whitney U tests.

|  | **No hypertrophic** | **Hypertrophic** | **p-value** |
| --- | --- | --- | --- |
| Tang (% CD3^+^) | 2.56 (1.70) | 2.35 (1.27) | 0.493 |
| EPC (% CD34^+^) | 10.51 (8.77) | 7.00 (6.58) | 0.060 |
| EC (% live gate) | 63.25 (27.48) | 77.57 (21.49) | 0.066 |
| CD4^+^CD28^null^ (% CD3^+^) | 16.82 (16.87) | 17.84 (8.55) | 0.940 |
| Non-classical (% MO gate) | 11.68 (7.67) | 11.02 (3.85) | 0.324 |
| Intermediate (% MO gate) | 6.28 (3.51) | 5.74 (3.70) | 0.960 |
| Classical (% MO gate) | 75.13 (6.38) | 77.40 (8.59) | 0.324 |
| ACE^+^ non-classical (% non-classical gate) | 53.78 (28.86) | 27.10 (23.41) | 0.394 |
| ACE^+^ intermediate (% intermediate gate) | 56.07 (24.31) | 48.80 (23.06) | 0.270 |
| ACE^+^ classical (% classical gate) | 3.17 (4.01) | 3.89 (5.35) | 0.776 |
| CD15^+^ LDG (% live gate) | 5.89 (8.01) | 4.21 (10.87) | 0.295 |
| CD16^+^CD14^low^ LDG (% CD15^+^ LDG) | 49.13 (46.80) | 36.79 (48.11) | 0.183 |
| CD16^-^CD14^-^ LDG (% CD15^+^ LDG) | 45.56 (49.87) | 57.27 (24.56) | 0.097 |

**Supplementary Table 2: Association between the levels of cell populations and time vintages from index procedure.** Associations were evaluated by Spearmans’ rank correlation tests and correlation coefficients (r) and p-values (p) are indicated.

|  | **Time course from index procedure to catheterization** | **Time course from index procedure to recruitment** |
| --- | --- | --- |
| Tang (% CD3^+^) | r=-0.126  p=0.344 | r=0.029  p=0.828 |
| EPC (% CD34^+^) | r=0.259  p=0.078 | r=0.269  p=0.058 |
| EC (% live gate) | r=-0.119  p=0.401 | r=-0.144  p=0.303 |
| CD4^+^CD28^null^ (% CD3^+^) | r=-0.309  p=0.112 | r=-0.329  p=0.080 |
| Non-classical (% MO gate) | r=0.006  p=0.966 | r=0.069  p=0.603 |
| Intermediate (% MO gate) | r=0.089  p=0.504 | r=0.099  p=0.452 |
| Classical (% MO gate) | r=0.060  p=0.652 | r=0.003  p=0.980 |
| ACE^+^ non-classical (% non-classical gate) | r=0.075  p=0.570 | r=0.205  p=0.116 |
| ACE^+^ intermediate (% intermediate gate) | r=0.214  p=0.103 | r=0.188  p=0.150 |
| ACE^+^ classical (% classical gate) | r=0.070  p=0.599 | r=0.162  p=0.216 |
| CD15^+^ LDG (% live gate) | r=-0.119  p=0.399 | r=-0.151  p=0.280 |
| CD16^+^CD14^low^ LDG (% CD15^+^ LDG) | r=0.092  p=0.516 | r=0.224  p=0.107 |
| CD16^-^CD14^-^ LDG (% CD15^+^ LDG) | r=-0.059  p=0.678 | r=-0.166  p=0.234 |
